# Dissociated Steroids

**DOI:** 10.1100/tsw.2007.97

**Published:** 2007-03-30

**Authors:** Matthew C. Catley

**Affiliations:** Respiratory Pharmacology, Airways Diseases, NHLI, Imperial College London, UK

**Keywords:** dissociated steroid, glucocorticoids, corticosteroids, inflammation, transrepression, transactivation

## Abstract

Glucocorticoids (GCs) are some of the most important drugs in clinical use today. They are mainly used to suppress disease-related inflammation and are widely used for the treatment of many inflammatory diseases including asthma and arthritis. However, GCs are also associated with debilitating side effects that place limitations on the long-term use of these drugs. The development of a GC with reduced side effects would allow more effective treatments for patients who require long-term suppression of inflammation. GCs exert their effects by binding and activating the GC receptor (GR). The activated receptor then binds GC response elements (GREs) in the promoter of genes, and activates transcription (transactivation) or interferes with the activation of transcription by inhibiting the transactivating function of other transcription factors, such as AP-1 and NF-ĸB (transrepression). Transrepression is believed to be responsible for the majority of the beneficial anti-inflammatory effects of GCs, whereas transactivation is believed to play a bigger role in the unwanted side effects of GCs. Compounds that can dissociate the transactivation function of GCs from the transrepression function may, therefore, have an improved therapeutic index. A number of these dissociated corticosteroids have been developed. *In vitro* assays using these compounds appear to show good dissociation. However, *in vivo*, the dissociation appears to be lost and these compounds still produce many of the side effects associated with conventional GCs. A better understanding of the molecular mechanisms behind GC-induced effects would allow the design of novel selective GR modulators with an improved therapeutic index.

## HISTORY OF CORTICOSTEROIDS AS A THERAPY

The Nobel prize for Medicine and Physiology was awarded to Edward C. Kendall, Tadeus Reichstein, and Philip S. Hench in 1950 "for their discoveries relating to the hormones of the adrenal cortex, their structure and biological effects" (http://nobelprize.org/nobel_prizes/medicine/laureates/1950/). While Kendall and Reichstein worked mainly on the isolation of adrenal hormones and the synthesis of cortisol, it was Hench, working at the Mayo Clinic, who demonstrated the dramatic effects of administering intravenous cortisone to patients with arthritis[1,2]. In 1949, Bordley (working at Johns Hopkins) showed that the adrenocorticotropic hormone (ACTH), which induces adrenal gland cortisol production, was effective in the treatment of five patients with eosinophilic asthma[3].

Corticosteroids are some of the most widely used and important drugs currently available, and are able to treat a broad range of clinical manifestations including asthma, arthritis, anaphylaxis, intercranial compression, systemic lupus erythematosus, and vasculitis[4,5]. In fact, corticosteroids are so important in the treatment of arthritis that some authors have divided the history of rheumatology into before cortisol (BC) and after cortisol (AC)[5]. However, it rapidly became apparent that steroids have pleiotropic effects on a number of different physiological processes. Indeed, many of the systemic adverse side effects that we now know are associated with excessive corticosteroid use were noted by Hench in his Nobel lecture[6,7]. These side effects and the high doses of systemic ACTH and cortisol employed led to a wave of patient phobia of corticosteroids[7].

## THE ANTI-INFLAMMATORY EFFECTS OF GLUCOCORTICOIDS

The inflammatory process is very complex, involving multiple mediators and cell types. The airway inflammation seen in asthma is used here as an example of the process and how glucocorticoids (GCs) have a unique anti-inflammatory effect. However, many of the basic features of asthmatic inflammation are similar in other inflammatory diseases. In asthma, the inhalation of an allergen causes degranulation of mast cells and the release of multiple preformed mediators that cause the early contraction of the airways. Many of these mediators probably activate the structural cells of the lung including the epithelium, airway smooth muscle, fibroblasts, and endothelial cells[4,8]. These cells are capable of producing multiple inflammatory mediators including chemokines (eotaxin), cytokines (IL-1β, TNFα, IL-4, IL-5), enzymes (iNOS, COX-2), adhesion molecules (ICAM-1, VACM-1), receptors (bradykinin $B_2$-receptors), and peptides (endothelin)[8]. The mediators then promote the infiltration of the airways by inflammatory cells, activation and survival of infiltrating immune cells, activation of structural cells, and the release of inflammatory mediators. Thus, therapies that target individual mediators have little chance of success. GCs are excellent anti-inflammatories because they are able to inhibit the production of many different inflammatory mediators including the examples given above[4]. GCs can also activate the transcription of anti-inflammatory genes including the inhibitor of kappa B (IκBα), annexin-1, secretory leukoprotease inhibitor (SLIPI), dual-specificity phosphatase 1 (DUSP1 or mitogen-activated protein [MAP] kinase phosphatase-1), glucocorticoid-induced leucine zipper protein (GILZ), and interleukin (IL)-10[9,10]. This ability of steroids to suppress the inflammatory process at multiple levels is probably why they are so effective in reducing inflammation.

## MECHANISMS OF GC ACTION

Synthetic or naturally occurring GCs are able to diffuse passively through the cell membrane where they bind the GC receptor (GR). Unliganded GR is located in the cytoplasm where it forms a complex with multiple proteins including p23; heat-shock proteins (HSPs) 90, 70, 56 and 30; and heat-shock cognate (HSC) 70[11,12]. Binding of ligand to the GR causes dissociation of this complex and GR translocation to the nucleus, where GR forms homodimers and modulates the expression of target genes[12,13]. Modulation of gene expression by the GR involves interaction with a wide range of cofactor proteins, and different combinations of these cofactors probably allow the GR to alter the activity of target genes in both positive and negative ways. For example, there is considerable recent data that indicate that GR recruits histone deacetylase (HDAC) activity to inflammatory gene promoters and this is thought to play a key role in the inhibition of inflammatory genes by activated GR. The activity of GR is also the target of a wide range of post-translational modifications including phosphorylation, sumoylation, acetylation, nitrosylation, and ubiquitinylation[14,15,16,17]. The exact functional role of these post-translational modifications is not entirely clear; for example, mutating all the phosphorylation sites has only a small effect on GR-mediated activation of the mouse mammary tumor virus (MMTV) promoter[17,18]. However, these phosphorylations may modulate the activity of GR in a promoter context specific manner by altering the interaction of GR with different cofactors at the promoter of target genes[17]. More information on the role of these post-translation modifications can be found in a recent review by Ito et al.[15].

## GR-MEDIATED SUPPRESSION OF TRANSCRIPTION

The binding of GR to negative GC response elements (nGREs) in the upstream promoter of a gene can suppress the expression of that gene[19]. Although this mechanism of gene repression may be important for some genes, nGREs have not been identified in the promoters of many inflammatory genes and thus do not appear to be very important to the anti-inflammatory effects of GCs[9]. Some of the side effects of GCs may be due to gene repression mediated through nGREs[4]. Liganded GR can interact with the promoters of inflammatory genes independently of DNA binding and suppress gene transcription[9]. This process of transrepression involves interaction of GR with other DNA-bound transcription factors, such as NF-κB, CBP, and AP-1[10]. GR either inhibits interaction between these transcription factors and the basal transcriptional machinery, or recruits corepressors or histone-modifying enzymes that terminate gene transcription[9]. There is now a body of evidence that suggests that GR promotes the recruitment of histone deacetylates (HDACs) to the promoters of inflammatory genes. These HDACs then deacetylate the histones and lead to a more closed chromatin structure that blocks the recognition promoters by positive transcriptional activators, such as NF-κB. This process prevents the activation of inflammatory genes[16,20,21]. Transrepression is historically considered the major mechanism by which GCs exert their anti-inflammatory effects[22]. However, there is also evidence to suggest that GR inhibits NF-κB activation by interfering with RNA polymerase II carboxy-terminal domain phosphorylation[23].

## ACTIVATION OF TRANSCRIPTION

GR activates transcription by binding to GREs as heterodimers and activating transcription of target genes through the recruitment to coactivators with histone acetylatase activity[12]. Although this may seem contradictory considering GR also promotes HDAC activity, it appears that GR-mediated transcriptional modulation is highly dependent on the promoter context and this would explain how GR acts as both an inhibitor and activator of transcription in the same cell. GR may also interact with coactivators, such as CBP, and play a role in activation of transcription independently of DNA binding[10]. Some of the anti-inflammatory effects of GCs may be mediated through GR activation of anti-inflammatory genes, such as SLIPI, DUSP1, or IκBα. Historically, the activation of these proteins by GR was considered to play a minor role in the anti-inflammatory effects of GCs. In contrast, the activation of transcription by GCs was believed to play a major role in many of the processes involved in the systemic side effects of GCs. However, it is becoming increasingly clear that GR-mediated activation of genes may have a more important role in GC-mediated anti-inflammatory effects.

## THE SIDE EFFECTS OF STEROIDS

Numerous side effects have been associated with steroid use, particularly the long-term use of oral steroids, and this has limited the usefulness of GC therapy for the treatment of chronic inflammatory diseases where systemic delivery is necessary.

Osteoporosis is one of the most predictable and debilitating complications of chronic GC treatment[24]. Corticosteroids modulate the normal process of bone mineralization and remineralization, which leads to a reduction in bone density. Another common side effect associated with GCs is disruption of the hypothalamic-pituitary-adrenal (HPA) axis, which causes a broad range of side effects including

Cushings syndrome, one of the best characterized side effects of excessive GCs[25,26]. GC disruption of the HPA axis also causes adrenal insufficiency and growth retardation[27]

Suppression of the immune system is the major therapeutically beneficial function of GCs. Unfortunately, this can lead to increased opportunistic infections, such as oral candidiasis[28]. Disturbed wound healing is also often observed in patients treated with systemic corticosteroids and appears to be caused by suppression of the initial inflammatory response that is required for efficient wound healing[29]. Corticosteroids also inhibit the production of growth factors, cytokines, matrix proteins, and proteases that are required for normal wound healing.

Other side effects associated with systemic GC use include muscle atrophy/myopathy, hyperglycemia due to disruption of glycemic control, hypertension, cataracts, and glaucoma.

For a more detailed discussion of the side effects associated with corticosteroids and their causes, see the review by Schacke et al.[29].

As indicated earlier, the majority of these side effects are thought to be mediated by transactivation of gene expression by GCs. However, it should be noted that this is not always the case and some side effects are caused by transrepression of gene expression.

## OVERCOMING THE SIDE EFFECTS OF GCs

The wide-ranging side effects caused by GCs have necessitated modification of GCs and treatment regimes to limit these side effects. One of the first such developments was the production of GCs with selectivity for the GR, rather than targeting both GR and Mineralocorticoid Receptor (MR) as is the case with the endogenous ligands. This diminishes many of the unwanted effects of GCs on the brain and kidney. However, side effects still remain a problem. Over the 50 years that GCs have been in clinical use, considerable knowledge of the most effective treatment regimes required to limit side effects and maximize benefits have been gathered. The principal of using “as much as necessary, but as little as possible” has been used to develop effective treatment of a number of diseases[7].

The development of GCs suitable for topical delivery has been successful for the treatment of disease where this delivery method is appropriate. In particular, the use of inhaled GCs for asthma treatment has reduced the morbidity and mortality associated with this disease substantially[4]. However, these inhaled compounds may still be associated with some systemic effects, such as growth retardation in children[9,29].

Another approach to limiting side effects is the development of so called “soft steroids”. These drugs are either inactive prodrugs that are enzymatically activated at their site of action, or active drugs that exert their desired effect at a specific site of action and are then broken down by esterification or other enzymic action to produce an inactive metabolite[30].

## DISSOCIATED STEROIDS

The nuclear receptor superfamily makes an attractive target for pharmacological modulation. Selective nuclear receptor ligands can be designed to act as antagonists, full agonists, partial agonists, or inverse agonists. In addition, compounds can be designed to activate only a subset of the functions induced by the endogenous ligand or which act as in a cell type–selective manner, the so-called selective nuclear receptor modulators (SNuRMs)[31]. Thus, it may be possible to design a GR ligand with reduced systemic side effects that retains all the beneficial anti-inflammatory properties. Since the anti-inflammatory properties of steroids have been associated with transrepression and many of the side effects with transactivation, it seemed likely that the dissociation of these two functions could provide a solely transrepressing GC with an improved therapeutic index. The importance of GCs to the treatment of inflammatory disease has generated considerable research interest in the development of these dissociated GCs and much of this work indicated that dissociation was possible.

Mutation of GR in the DNA binding domain prevents transactivation, but leaves transrepression of AP-1 intact, demonstrating that these functions can be separated[32]. Transactivation by GR was later shown to be dependent on GR dimerization, whereas transrepression was not affected by mutations that prevent dimerization[33]. The transrepression was also dependent on the presence of a ligand binding domain, demonstrating that transrepression is ligand dependent[33]. The first dissociated steroid, RU24858, was developed and appeared to be able to promote transrepression without transactivation of GR-dependent genes *in vitro*[34]. The generation of $GR^{dim/dim}$ mice, a A458T mutation within the GR dimerization loop that prevents GR DNA binding and GR transactivation of classical GREs, but leaves the transrepressive DNA binding independent properties of GR intact, further indicated that these processes can be separated[22].

Vayssiere et al. showed that the dissociated steroid RU24858 could transrepress AP-1–dependent transcription with a similar potency to dexamethasone, but its ability to promote GRE-dependent transcription was considerably reduced. RU24858 was also shown to inhibit IL-6 transcription through inhibiting NF-κB activity[34,35]. This compound was also shown to retain anti-inflammatory properties both *in vivo* and *in vitro*. However, subsequent work by Belvisi et al. indicated that this compound was not completely dissociated *in vivo* and still caused weight loss, loss of bone density, and thymus involution[36]. Other parameters were not tested, such as diabetes mellitus, glaucoma, opportunistic infection, and behavioral change. However, this study indicated that RU24858 did not have a greater therapeutic index than conventional GCs[36]. A recent study indicated that RU24858 can alter the osteoprotegerin/receptor activator of nuclear factor κB (OPG/RANKL) ratio in a manner likely to cause osteopenia, although the effect is not as pronounced as with conventional GCs[37]. This process is dependent on the transrepression of OPG production and demonstrates that not all side effects will be lost with dissociation of transactivation and transrepression[37]. Additional studies have indicated that the dissociation of transactivation and transrepression by RU24858 may be cell type specific and this may explain the poor separation of these processes *in vivo*[38].

A second dissociate GR ligand has recently been described. ZK216348 has similar anti-inflammatory effects to prednisolone in *in vivo* models of inflammation[37,39]. Moreover, this compound was shown to have reduced side effects in terms of OPG/RANKL ratio, skin thinning, thymus and spleen weight reduction, tyrosine aminotransferase (TAT) induction, and hyperglycemia[37,39]. However, ZK216348 still suppresses the HPA axis and this is thought to be mediated through transrepression of corticotrophin-releasing hormone, meaning that this side effect may be resistant to dissociation of transactivation and transrepression[39]. The main problem with ZK216348 is that although it shows improved therapeutic index as a GR agonist, it also acts as an antagonist for MR and progesterone receptor (PR) and this may cause additional side effects[39].

AL-438, a recently described nonsteroidal GC ligand, is sometimes classified as a dissociated steroid[40,41]. However, it is not dissociated along the transactivation/transrepression function since it is still capable of activating transcription from a GRE-dependent promoter in HepG2 cells with similar efficacy as, but less potency than, prednisolone[40]. AL-438 is a potent anti-inflammatory in *in vivo* models of acute and chronic inflammation, and appears to have several benefits over prednisolone in terms of side effects[40,41]. Inhibition of markers of GC-mediated bone destruction are not as efficiently reduced by AL-438 as they are by prednisolone[40]. Also, RANKL is not induced by AL-438 as highly as it is by prednisolone, indicating a less-pronounced effect on the RANKL/OPG ratio[37]. AL-438 does not induce hyperglycemia in rats to the same extent as prednisolone, indicating that it is affecting glucose metabolism to a lesser extent[40]. The mechanism of AL-438 is slightly different than the other dissociated steroids discussed in that it does not dissociate between transactivation and transrepression. It is believed that it achieves its dissociation by differential recruitment of cofactors. Prednisolone stimulates GR interaction with peroxisome proliferators activated receptor-γ coactivator-1 (PGC-1), a cofactor required for hepatic glucose production, and glutamate receptor interacting protein 1 (GRIP-1), which is a cofactor involved in transcriptional repression of inflammatory genes[40]. In contrast, AL-438 promotes interaction of GR with only GRIP-1 and thus is unable to inhibit inflammatory gene transcription, but not promote hyperglycemia in the same way as prednisolone[40]. Thus, AL-438 acts more as a selective GR modulator (SGRM) rather than a dissociated steroid. Since some of the side effects associated with GCs are caused by transrepression of gene transcription, it may be that SGRM-type compounds may have more promise than dissociated steroids. In all, the evidence suggests that AL-438 may have an improved therapeutic index over prednisolone, although a number of other side effects associated with GCs needs to be examined before definite conclusions can be made.

A compound of plant origin has recently been shown to act as a dissociated corticosteroid. The compound, 2-(4-acetoxyphenyl)-2-chloro-*N*methyl-ethylammonium chloride (Compound A), is a stable analogue of the hydroxyl phenyl aziridine precursor found in the Namibian shrub *Salsola tuberculatiformis* Botschantzev[42]. It binds the GR with equal affinity to dexamethasone, but is not able to induce transactivation of a GRE reporter gene. *In vivo* Compound A is as effective at treating acute inflammation as dexamethasone, but does not induce hyperglycemia[42]. There is only one report regarding this compound at present and further work is required to determine its side effect profile more fully.

A number of other dissociated steroids are in development, but it is presently not clear if these have any benefits over current compounds, as their *in vivo* side effects have not been determined[43,44].

The problem with the majority of these compounds is that they show good separation of transactivation or transrepression in cell-based assays using simple GRE-based promoters, or by looking at a small number of biomarkers in a limited number of cell types, whereas *in vivo*, the effects seem to be less clear cut with limited loss of side effects. This may be because these compounds are not fully dissociated and inhibit a subset of GR-regulated genes or are not capable of activating the transcription of simple GRE base reporter genes. The promoters of genes regulated by GR are often more complex than these simple reporter gene constructs, and multiple factors and cofactors are involved in the initiation of transcription by these promoters. An added level of complexity is the differential expression of some cofactors between cell types. Thus, these compounds may only show full dissociation with respect to a subset of GR-regulated genes and in a limited number of cell types. Future characterization of potential, dissociated steroids should look at global gene expression using gene arrays to determine true dissociation. *In vivo* studies are also vital to determine the effects of these compounds across a range of tissues.

## TRANSACTIVATION MAY BE VITAL TO THE ANTI-INFLAMMATORY EFFECTS OF GCs

The poor therapeutic index of dissociated compounds could be because transactivation is required for the full repression of inflammatory gene transcription. Transrepression of NF-κB is believed to be a major mechanism by which GCs reduce inflammatory gene transcription. However, in cells that are sensitive to GC inhibition of gene transcription, such as pulmonary epithelial cells and human airway smooth muscle cells, GCs cause only a 40% inhibition of NF-κB–dependent reporter gene activity and have no effect on NF-κB DNA binding[45,46,47]. Moreover, the expression of some genes is highly dependant on NF-κB activity, but not responsive to GCs, a good example of which is ICAM-1[45,48]. Thus, these data indicate that transrepression of NF-κB is not the primary mechanism of GC-mediated repression of inflammatory gene transcription in pulmonary epithelial cells and airway smooth muscle. The regulation of some inflammatory genes by GCs appears to involve post-transcriptional or translational mechanisms[12,49]. For example, GCs appear to inhibit IL-1β, IFNβ, GM-CSF, COX-2, IL-8, and IL-6 gene expression by destabilization of their respective mRNAs[49,50,51,52,53,54,55]. Investigation of this effect on mRNA stability indicates that it is dependent on both transcription and translation since blocking these processes with actinomycin and cycloheximide, respectively, blunts the repression of these inflammatory mediators by dexamethasone[49,56]. Steroids are believed to influence mRNA stability by reducing p38-mediated stabilization of mRNA[55]. AU-rich elements (ARE) in the 3’ untranslated region of mRNAs appears to be essential for p38-mediated stabilization[57]. The AREs are bound by a host of proteins that are possible downstream targets for p38 phosphorylation[57]. However, the precise target for p38-mediated stabilization is not clear. The mechanism of dexamethasone-mediated destabilization is believed to involve the transcriptional up-regulation of DUSP1, which dephosphorylates and deactivates MAP kinases in the p38, JNK, and ERK signaling pathways[58,59,60]. In agreement with this, a recent study demonstrated that knockout of DUSP1 prevents dexamethasone-mediated inhibition of p38, JNK, and ERK phosphorylation[61]. This study also demonstrated that DUSP1 is required for the repression of some inflammatory markers by dexamethasone. Interestingly, the level of DUSP1-mediated repression of inflammatory markers by dexamethasone varies between markers, IL-1α repression is highly dependent on DUSP1, whereas COX-2 is only partially and IL-12 is not dependent at all. DUSP1 expression is induced by dexamethasone in $GR^{dim/dim}$ mice, indicating that dimerization is not required for transactivation of this gene[61]. Thus, transactivation of DUSP1 may be essential for some of the anti-inflammatory effects of GCs and selection of compounds that favor transrepression of gene expression may reduce some anti-inflammatory effects of GCs. This study also raises questions regarding the need for dimerization of GR in GR-mediated transactivation of gene expression. Another recently published study also demonstrated that the dissociated steroid RU24858 is still capable of transactivating DUSP1 expression in A549 cells. The authors suggest that RU24858 activates transcription from nonclassical GREs where GR dimerization is not required[56]. Thus, the design of novel selective steroids should take into account the role of transactivation of anti-inflammatory proteins such as DUSP1.

## SUMMARY

The demonstration that GR-mediated transactivation and transrepression could be separated created considerable interest in the possibility of creating novel GR ligands with improved therapeutic index. However, in the ~9 years since the concept of dissociation was shown to be possible, very few genuine dissociated ligands have been produced. Those that have been reported in the literature have shown little benefit over conventional steroids in terms of reduced side effects or have poor specificity particularly when tested in the more complex *in vivo* environment. Further work is required to determine if other compounds, such as AL-438 or Compound A, have a better therapeutic index for treating inflammatory diseases. However, there is also a growing body of evidence to suggest that transactivation of genes such DUSP1 or annexin1 may have an important role in some of the anti-inflammatory effects of steroids. If this is the case, then the design of selective and dissociated steroids may need to be rethought.

Recently, it was demonstrated that very subtle changes in GR ligand chemistry can have profound effects on the transcriptional regulatory activity of GR in cells. Moreover, this study indicates that it may be possible to design GR ligands that modulate the expression of a distinct subset of GR-regulated genes[62]. In addition, it is becoming more widely accepted that GR exists as multiple splice variants and it is possible that GR has more than one ligand *in vivo*[63,64]. The role of splice variant–alternative GR ligands needs to be investigated further in order to determine how GR activity is regulated *in vivo* in diverse tissues. It is thought that different GR ligands can create distinct “surfaces” on the GR that modulate its interaction with the DNA or with other cofactors[62]. Thus, a more complex compound profile may be required to improve the anti-inflammatory therapeutic index of GR ligands. For example, it may be that ligands can be designed to increase interaction between GR and repressive cofactors, such as the HDACs, or it may be possible to target GR to particular promoters based on the type of GRE or presence of other transcription factors. Alternatively, it may be possible to design cell- or tissue-specific GR ligands. Therefore, further work is required to define how GR modulates gene expression at the promoter level, and if there are different mechanisms involved in GR-mediated side effects and GR-induced anti-inflammatory actions. This would then allow more efficient screening for compounds with the most beneficial profile of anti-inflammatory effects. However, the possibility that this will be achieved by dissociation of transactivation and transrepression seems unlikely.
